# Surfing the Protein-Protein Interaction Surface Using Docking Methods: Application to the Design of PPI Inhibitors

**DOI:** 10.3390/molecules200611569

**Published:** 2015-06-23

**Authors:** Rushikesh Sable, Seetharama Jois

**Affiliations:** Department of Basic Pharmaceutical Sciences, School of Pharmacy, University of Louisiana at Monroe, 1800 Bienville Drive, Monroe, LA 71201, USA; E-Mail: sable@warhawks.ulm.edu

**Keywords:** protein-protein interactions, docking, protein docking, hot spots, virtual screening, drug-like molecules

## Abstract

Blocking protein-protein interactions (PPI) using small molecules or peptides modulates biochemical pathways and has therapeutic significance. PPI inhibition for designing drug-like molecules is a new area that has been explored extensively during the last decade. Considering the number of available PPI inhibitor databases and the limited number of 3D structures available for proteins, docking and scoring methods play a major role in designing PPI inhibitors as well as stabilizers. Docking methods are used in the design of PPI inhibitors at several stages of finding a lead compound, including modeling the protein complex, screening for hot spots on the protein-protein interaction interface and screening small molecules or peptides that bind to the PPI interface. There are three major challenges to the use of docking on the relatively flat surfaces of PPI. In this review we will provide some examples of the use of docking in PPI inhibitor design as well as its limitations. The combination of experimental and docking methods with improved scoring function has thus far resulted in few success stories of PPI inhibitors for therapeutic purposes. Docking algorithms used for PPI are in the early stages, however, and as more data are available docking will become a highly promising area in the design of PPI inhibitors or stabilizers.

## 1. Introduction

Rational drug design has revolutionized the pharmaceutical industry with the idea that drugs can be designed or engineered according to an identified protein or DNA target. This is an efficient alternative method in the drug design and discovery areas as opposed to screening thousands of samples extracted from natural products for therapeutic purposes. However, such a rational design of drugs is knowledge-based and requires an understanding of the intermolecular forces involved, as well as an understanding of protein structure and function. Although compounds are created based on the structure of the protein that is targeted, once a basic template is developed several possible combinations of the functional groups that are spatially separated by a particular distance must be investigated to obtain a lead compound to be optimized. In other words, designed compounds have to be optimized for drug-like properties in three-dimensional space. This type of optimization necessitates knowledge of the 3D structures of the protein receptors involved as well as the conformational space of the ligand drug [1]. This method of optimization has gained momentum as the number of available 3D structures of proteins has increased rapidly in the last two decades. Along with advances in genomics and proteomics, the methods to obtain the X-ray crystal or NMR-based structure of proteins have enabled new protein targets that have therapeutic potential. With detailed information about the binding site of a protein receptor available, computational methods such as docking have gained importance in optimizing drug-like compounds. Such computational methods were established in the 1980s [2] for the drug discovery process; however, there were several limitations. With advances in high-speed computers, development of efficient parallel processing algorithms, and availability of high-resolution 3D structures of receptor proteins, docking methods have become more reliable for optimizing the compounds for therapeutic purposes. In addition, docking methods are also used for analysis of drug metabolites using the structure of cytochrome p450 isoforms [3].

Drug creation involves several stages, starting from the design of a compound to lead identification (hit-to-lead), optimization, preclinical and toxicology reports for a New Drug Application (NDA), and final approval from the Food and Drug Administration (FDA) [4,5]. As drug-like molecules are taken to the next level in each stage described above, the cost of optimization increases significantly [6]. As the overall cost of creating a drug and bringing it to the market has skyrocketed, computational methods of creating drug-like molecules have gained importance. Most of the process of screening using docking occurs in the early stages of drug discovery. Typically, high-throughput screening (HTS) of compounds is used in the drug creation process, even in the rational drug discovery process, to find a lead compound. HTS involves screening of compounds using biochemical or cellular methods to determine the optimum pharmacological activity. For such methods, the proposed number of compounds is large, and synthesizing and evaluating such large numbers is a difficult task. Docking *in silico* methods provide a faster and less expensive way of screening compounds [7]. However, there are few successful examples of drug design using such methods. For the past two decades, computational and docking methods have gained popularity in different stages of drug design [1,8,9]. In the drug creation process, docking is a virtual screening method for possible target identification and lead optimization. Docking involves prediction of ligand orientation and different possible conformations within the receptor cavity or near the protein surface. In its simplest form, the binding cavity in the receptor or protein surface of the receptor protein is assumed to be rigid with only the ligand allowed to be flexible. The overall docking process involves two steps: namely, a conformational search of the ligand molecule within the defined grid box near the binding surface of a protein to represent different possible conformations and scoring, where different possible conformations generated in different orientations are scored based on energy function [10,11]. Based on the scoring method used, ligand conformations are arranged from lowest to highest energy order with the lowest energy conformers considered as a possible “pose” of the molecule in the bound form with the receptor. Depending on the application, users can employ an exhaustive search method and then optimize the scoring or search with optimized values and use the robust scoring function [12,13]. In most cases, searching methods are time-consuming, and searching for all possible conformations and orientations of a molecule on the receptor surface is an impossible task.

## 2. Protein-Protein Interactions

Cells communicate with one another via protein-protein interactions. All of the physiological processes of life are controlled via interactions of different proteins that are well regulated. In an organism, PPI form a huge complex network known as an “interactome,” which contributes significantly to the biological processes that are carried out in that organism [14]. It is estimated that there are nearly 650,000 interactions that regulate human life, and any deregulation of this process leads to a disease state [15]. These interactions control signal transduction, immune response, transcription, *etc.* Hence, among these PPI, at least a sizable number of proteins can be used as drug targets [16,17,18]. Many proteins interact in an obligatory fashion, maintaining a stable interaction for a longer period of time whereas some protein-protein interactions are transient. The affinity of PPI varies, depending on the type of interaction and signaling needed; this affinity can vary from millimolar to picomolar [19]. Although their affinity varies over a wide range, all PPI maintain a high degree of specificity for their partners, including many proteins that exhibit specificity for multiple partners [20]. How one protein can form specific interactions with different partners either simultaneously or separately depends on the nature of the interaction surface. In other words, the “molecular recognition” is a key concept in PPI, its affinity, specificity, and selectivity. A detailed knowledge of the interaction surfaces of proteins and their energetics is necessary to understand the regulatory mechanisms of biochemical pathways with the goal of modulating or blocking these pathways for therapeutic purposes using drug-like molecules. The analysis of 3D structures of many protein complexes and the nature of interfaces forming PPI has revealed that the contact surface involved in PPI is relatively large, ranging from 1000 to 4000 Å^2^. It is reported that standard-sized interfaces are 1200 to 2000 Å^2^ [21]. Smaller interfaces of 1150–1200 Å^2^ size normally constitute short-lived and low-stability complexes, and large surfaces ranging from 2000 to 4600 Å^2^ are observed between proteases and particular inhibitors and between G-proteins and other components of the signal transduction system [22]. In comparison, protein-small molecule interaction surfaces have an area of 300 to 1000 Å^2^. In addition to this, surfaces of PPI are generally flat and lack the grooves and pockets that are present at the surfaces of proteins that bind to small molecules [23,24,25,26]. Although a description of PPI was known more than two decades ago, because of the large surface area of PPI, small molecules that were targeted were unsuccessful. It was viewed that targeting PPI had high risk and such targets were considered “undruggable.” However, this concept was challenged, and now there are drugs on the market [18,27,28] and drug-like candidates that target PPI are in clinical trials [29]. Since PPI surfaces are relatively flat and the docking methods applied use probe atoms or ligand atoms on the flat surface, we use the word “surfing” for identification of hot spots and ligand-binding mode at PPI interface.

The most significant contribution to understanding the PPI surface comes from structural biology via X-ray crystallography or NMR as well as mutational studies. Detailed 3D structure analysis of PPI revealed that PPI interfaces can form a continuous epitope that often forms a single secondary structure or discontinuous epitope originating from multiple secondary structures. These continuous or discontinuous epitopes form small pockets and groove-like structures that together form a binding site for proteins. Protein-protein interaction surfaces are generally hydrophobic in nature. This was assessed by measuring the area of accessible surface on the protein surface that forms the interface region of partner proteins that becomes inaccessible to solvent due to protein-protein contacts. It is known that the PPI interface area is large; however, only certain hydrophobic spots contribute to the free energy of binding and help to hold the two proteins together. Such regions on PPI interfaces that contribute more to the binding energy are called hot spots. Hot spots account for less than 50% of the contact area of PPI. A region of protein surface is called a hot spot when replacement of an amino acid residue by alanine in that spot lowers the free energy of binding by at least 2 kcal/mol [30]. The hot spots have a core region and rim region with more accessible rim region residues surrounding the more buried core region residues. The amino acid composition in the rim region is similar to that of the rest of the protein surface, whereas the core region contains aromatic residues [31,32,33]. Analysis of the amino acid composition of hot spots shows that some residues are found more frequently in hot spots, namely, Tyr, Trp, and Arg [34]. The hot spots are surrounded by energetically less important residues that probably separate/prevent bulk water from hot spots. The presence of these hot spots provides an opportunity to target PPI with therapeutic agents because compounds that are designed to interact with hot spots should prevent or block PPI since a large part of the binding energy contributes to interaction in these areas. For hot spots that are discontinuous and distributed over an area, relatively large molecules such as peptides have been designed to prevent PPI. In terms of amino acid functional groups, Trp, Tyr, Leu, Ile, Phe, and Arg are frequently found in PPI hot spots [34]. Among these, Trp has a hydrophobic surface and contributes to π-interactions that contribute to binding energy. Apart from this, Trp can form hydrogen bonds with ligand molecules without the introduction of water at the PPI site. Tyr also has a hydrophobic surface that can produce π-interactions and can form hydrogen bonds. Arg can form up to five hydrogen bonds and salt bridges as well as hydrophobic interactions with its long side chain. Between the amino acids Leu and Ile, Ile seems to be preferred at PPI [21]. While there is much detailed structural information available about the PPI, there are still many challenges in the design of PPI inhibitors. Some of these challenges have been discussed in reviews [26,32,35,36,37,38,39,40]. Recent literature suggests that the modulation or inhibition of PPI has advanced rapidly in the last few years [41,42,43,44,45,46,47,48,49,50,51].

## 3. Identification of Hot Spots on Proteins and Docking in PPI

**Figure 1:**
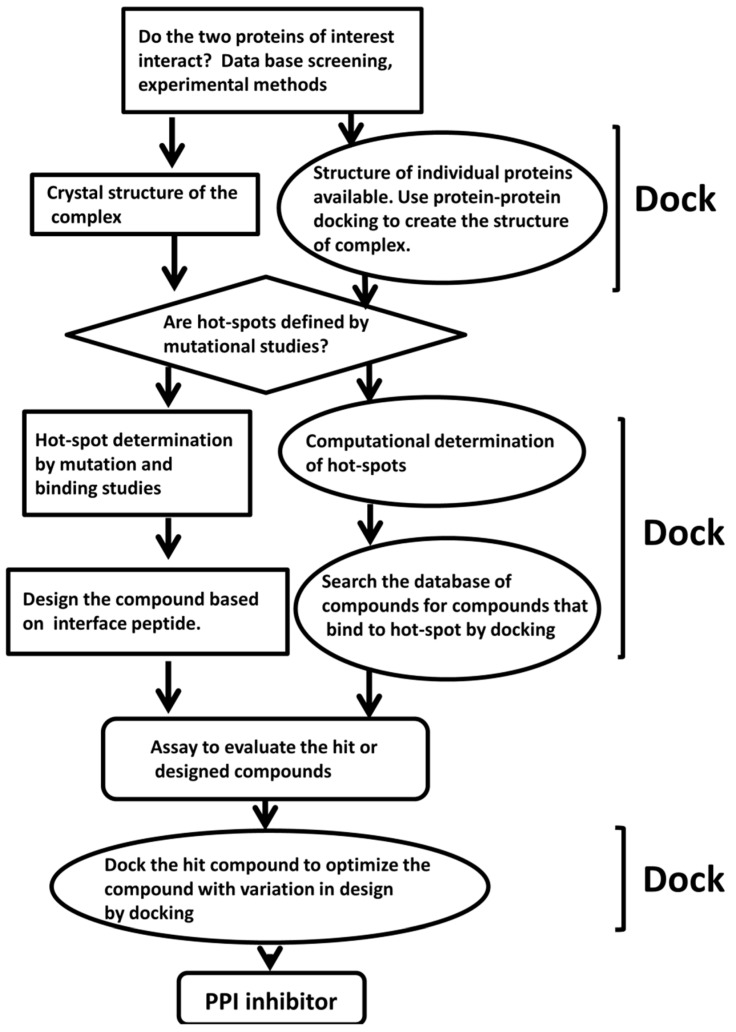
A schematic diagram for the design of PPI inhibitors and docking methods used at different stages of the design.

Docking methods are used in several stages during the design of PPI inhibitors (Figure 1). Even before docking methods are applied to find the interface residues, the first question asked is whether the two proteins of interest interact with one another or not [52].

Experimental procedures such as yeast two-hybrid method, co-immunoprecipitation, pull-down assay, and protein chip- or mass spectrometry-based assays can be used to identify the protein partner interactions. Several PPI databases are available in the literature (Table 1). Advances in molecular biology and proteomics methods as well as interest in PPI in many research laboratories have led to large amounts of data being produced. Genome-scale analysis of PPI in organisms such as bacteria, yeast, worm, fly, and human have produced enormous amounts of data [53]. PPI network data generated has been used to elucidate biochemical pathways [54] as well as protein function [55] and disease associated with deregulation of these pathways [56]. However, the data generated from PPI network may or may not be successful in providing information about underlying PPI. This is because analysis of the data and the results obtained depend on the technique used. Wodak *et al.* [57] have discussed the limitations of the databases and the fact that they are highly dependent on the detection method used, the error generated, and the challenges faced by PPI network databases. The same data analyzed by different computational procedures yield poor overlap in the results obtained which is a major concern. They suggest that the poor overlap between the PPI datasets seems to arise from the fact that quite a large fraction of data about interactions comes from non-functional interactions. They suggest that future studies should use approaches that incorporate identification of the functional portions of the interactome as well as characterizing its non-functional complement.

**Table 1:** List of protein-protein interaction databases and servers.

Database	Website	Reference
2P2I	http://2p2idb.cnrs-mrs.fr/	[58,59]
PrePPI	http://bhapp.c2b2.columbia.edu/PrePPI	[60]
STRING	http://string-db.org/	[61,62]
IBIS	http://www.ncbi.nlm.nih.gov/Structure/ibis/ibis_help.shtml#whatisIBIS	[63,64]
PIPS	http://www.compbio.dundee.ac.uk/www-pips/	[65,66]
PredUS	https://bhapp.c2b2.columbia.edu/PredUs/	[67,68]
DIP, LiveDIP	dip.doe-mbi.ucla.edu/ldipc/tmpl/livedip.cgi	[69]
BIND	http://www.bindingdb.org/bind/index.jsp	[70]
MPact/MIPS	http://mips.helmholtz-muenchen.de/proj/ppi/	[71]
YPD and WormPD	https://portal.biobase-international.com/build_ghpywl/idb/1.0/html/bkldoc/source/bkl/proteome/proteome_wormpd_intro.html	[72,73]
MINT	http://mint.bio.uniroma2.it/mint/Welcome.do	[74,75]
IntAct	http://www.ebi.ac.uk/intact/	[76]
BioGRID	http://thebiogrid.org/	[77]
HPRD	http://www.hprd.org/	[78,79]
ProtCom	http://www.ces.clemson.edu/compbio/protcom	[80,81]
3did, Interprets	http://3did.irbbarcelona.org/	[82,83]
Pibase, ModBase	http://modbase.compbio.ucsf.edu/pibase/introduction.html	[84,85]
CBM	http://www.cazy.org/Carbohydrate-Binding-Modules.html	[86,87]
SCOPPI	http://scoppi.biotec.tu-dresden.de/scoppi/	[88,89]
iPfam	http://www.ipfam.org/	[90,91]
InterDom	http://interdom.i2r.a-star.edu.sg/	[92]
DIMA	http://webclu.bio.wzw.tum.de/dima/	[93]
Prolinks	http://prl.mbi.ucla.edu/prlbeta/	[94]

Before extensive docking studies, hot-spot identification studies, and design of PPI can begin, experimental methods must be used to show that the two proteins of interest interact with one another or to identify the partner of a known protein of interest. If the structure of the complex of protein partners is available along with mutational data, hot spots are readily available for docking of designed drug-like molecules to one of the proteins. However, if the structure of the complex is not available but the structures of individual proteins are available along with molecular biology data on mutation of residues and binding, protein complexes may be built to identify the binding interface. In the first step when the structures of monomers of interacting partners are available, docking methods are used to determine the complex structure of the protein. There are two types of docking used for protein-protein docking: (1) Template-based docking, where the structures of individual proteins are manually docked using the template structure of a dimer in the same homologous family. This method is fast and simple in the sense that no automatic docking and scoring algorithms are used. The generated structure of the complex based on a template is minimized to obtain the docked conformation; (2) Template-free docking, in which the structures of monomers are docked using docking algorithms with or without the support of experimental data. Several protein-protein docking methods are available. Some available databases (Table 1), docking methods (Table 2) that have been used for several years, and scoring/refining methods (Table 3) that have been improved over time are provided. An updated list of all these methods and functions is available at http://www.vls3d.com/index.php/links/bioinformatics/protein-protein-interaction/protein-protein-docking.

**Table 2:** List of protein-protein docking methods.

Method	Website	Type	Reference
HADDOCK	http://www.bonvinlab.org/software/haddock2.2/haddock.html	Online	[95]
pyDOCK*	http://life.bsc.es/pid/pydock/	Software	[96]
Cell-Dock	http://mmb.pcb.ub.es/~cpons/Cell-Dock/	Software	[97]
KBDOCK	http://kbdock.loria.fr/	Online/Database	[98]
F2DOCK	www.cs.utexas.edu/~bajaj/cvc/software/f2dock.shtml	Online	[99]
BiGGER	http://centria.di.fct.unl.pt/~ludi/bigger.html	Software	[100]
FRODOCK	www.chaconlab.org/methods/docking/frodock	Online	[101]
DOCK/PIERR	http://clsb.ices.utexas.edu/web/dock.html	Online	[102]
ZDOCK	http://zdock.umassmed.edu/	Online	[103,104]
PI-LzeRD	www.kiharalab.org/proteindocking/pilzerd.php	Software	[105]
ATTRACT	http://www.t38.ph.tum.de/index.php?id=88	Software	[106]
Swarmdock	http://bmm.cancerresearchuk.org/~SwarmDock/	Online	[107]
PruneandProbe	http://chembio.iisc.ernet.in/prune/	Online	[108]
3D-GARDEN	www.sbg.bio.ic.ac.uk/~3dgarden/	Online	[109]
LIGIN	http://swift.cmbi.ru.nl/gv/start/index.html	Software	[110]
Smoothdock	http://structure.pitt.edu/servers/smoothdock/	Online	[111]
DOT	www.sdsc.edu/CCMS/DOT/	Software	[112]
RosettaDock	http://graylab.jhu.edu/docking/rosetta/	Online	[113]
Molfit	www.weizmann.ac.il/Chemical_Research_Support/molfit/	Software	[114]
Hex	http://hex.loria.fr/hex.php	Online	[115]
ESCHER-NG	www.ddl.unimi.it/escherng/index.htm	Software	
GRAMM	http://vakser.compbio.ku.edu/resources/gramm/grammx/	Online/Software	[116]
ClusPro	http://cluspro.bu.edu/login.php	Online	[117,118]
SEQMOL	www.biochemlabsolutions.com/FASTAandPDB.html	Software	[119]
Datasets	www.lgm.upmc.fr/CCDMintseris/	Software/Dataset	[120]
UDOCK	www.udock.fr/	Software (Windows)	[121]
FireDock	http://bioinfo3d.cs.tau.ac.il/FireDock/	Online	[122,123]
FTDock	www.sbg.bio.ic.ac.uk/docking/ftdock.html	Software	[124]
HingeProt	www.prc.boun.edu.tr/appserv/prc/hingeprot/usage.html	Software	[125]
ICMpro	www.molsoft.com/icm_pro.html	Software	[126]
PatchDock	http://bioinfo3d.cs.tau.ac.il/PatchDock/	Online	[127]
SymmDock	http://bioinfo3d.cs.tau.ac.il/SymmDock/	Online	[127]

**Table 3:** List of scoring, and refining methods/software and servers.

Method	Website	Type	Reference
FunHunt	http://funhunt.furmanlab.cs.huji.ac.il/	Online	[128]
Fiberdock	http://bioinfo3d.cs.tau.ac.il/FiberDock/	Online	[129,130]
TACOS	http://zhanglab.ccmb.med.umich.edu/TACOS/about.html	Online	[131]
SPA-PP	https://www.dl.dropboxusercontent.com/u/1865642/Optimization.cpp.	Software	[132]
FastContact	http://structure.pitt.edu/servers/fastcontact/	Online	[133]
CONSRANK	www.molnac.unisa.it/BioTools/consrank/	Online	[134]
FILTREST 3D	www.genesilico.pl/software/stand-alone/filtrest3d	Software	[135]

We discuss few methods of protein-protein docking here. ZDOCK is a software developed by Chen *et al.* [136] in which proteins were treated as rigid objects and 6-dimenional rotational and translational degrees of freedom were explored. For this initial stage, surface complementarity, electrostatic complementarity, and desolvation parameters were used to search for different conformations using the fast Fourier transform method (FFT). After an initial search, a number of conformers were identified and ranked according to the scoring criteria used. In the second stage, these conformers were re-ranked, and energy minimization was performed to refine the structures. Later, to improve the performance of the docking, ZDOCK 3.0 was used. It has a scoring function that includes shape complementarity, electrostatics, and a pairwise atomic statistical potential developed using contact propensities of transient protein complexes [103,137]. As an example to show the efficiency of the computational method developed, the authors applied the method to predict the structure of the yeast interactome using a large supercomputing cluster [103,138]. The method was efficient in terms of the use of computational resources. ZRANK, a scoring algorithm that [139] relies on the usage of a combination of three atom-based terms, *i.e.*, van der Waals, electrostatics, and desolvation, was used to rank the structures.

Protein-protein docking methods developed use a similar general approach in which one protein is fixed in space and the second is rotated and translated around the first one. For each new configuration, the energy of interaction is calculated based on terms such as surface complementarities, electrostatic interactions, van der Waals interaction, and additional terms depending on the method developed. The calculated configurations are given a score based on energy functions used. One of the drawbacks of these methods is that it is almost impossible to search every possible rotation and translation for two interacting objects, and the search through the entire conformational space of the complex geometry makes the calculation very time-consuming, seldom resulting in a unique solution. A docking method that incorporates experimental data to dock the two protein structures was developed by Domingues *et al.* [140,141]. In the high ambiguity driven docking approach (HADDOCK), the user must provide information obtained from biochemical and chemical shift perturbation data from NMR titration, as well as mutagenesis experiments [140]. Based on the information input on the interacting residues, ambiguous interaction restraints (AIRs) are introduced during the docking to arrive at the most possible orientation of the two proteins. Structures are ranked according to their intermolecular energy, that is, the sum of electrostatic, van der Waals, desolvation, and AIR energy terms after the docking calculations are completed.

A new computational method that uses an assembly of structures of molecules that can fit into an electron density map data generated by cryo-electron microscopy (cryo-EM) electron density maps was described by de Vries *et al.* [142]. The method is based upon ATTRACT, an atom-based protein-protein docking program [142,143,144]. Compared to other protein docking methods, this technique is unique. Since the cryoelectron microscopy data does not provide high-resolution structures of proteins at the atomic level, the starting model uses a coarse-grained force field where proteins are represented by up to four (pseudo-)atoms per amino acid. The protocol uses an atom-to-grid cryo-EM fitting, using ATTRACT’s coarse-grained atom model; the generated models are energy-minimized, and these generated structures are mapped onto the electron density map. In a two-step model, the initial models are fitted using low-resolution data and are then re-scored using a gradient vector matching algorithm to generate models of protein complexes. The models with the best fit with the electron density map and the best scored models are refined to obtain a higher resolution model that is optimized using the ATTRACT force field.

Since different docking programs developed by different researchers around the world use different criteria for scoring based on the need and the problem encountered, a general assessment method for results of docking was established to compare the quality of docked protein complex structures. Performances of docking algorithms are compared biannually in the Critical Assessment of Predicted Interaction competition (CAPRI) [145] and are evaluated against larger protein docking benchmarks [146,147,148,149]. The model of the protein complex generated using docking method can be evaluated with CAPRI criteria. It provides information about how reliable and accurate the model is likely to be compared to the experimentally generated structures.

### 3.1. Identification of Hot Spots and Druggability

One of the most important steps in PPI design is identification of hot spots using computational or experimental methods. Alanine scanning is a widely used experimental method for hot-spot identification. Apart from this, NMR and X-ray crystallographic methods along with mutational data are used to define hot spots in 3D structures of protein complexes. These hot spots are suitable for binding to a variety of small molecules and, hence, can be used as “druggable” hot spots with high hit rates [150,151]. There are several computational methods available to identify the hot spots, and some of these methods are reviewed by Morrow and Zhang [152]. A list of methods/software that are available free for academic users to determine hot spots on protein surfaces and the websites of interest are provided in the Table 4. Some of the hot-spot determination methods use docking algorithms to find the hot spot; we will provide a brief description of those methods. The method developed by Brenke *et al.* uses the data obtained from structure-activity relationships (SAR) by NMR and multiple solvent crystal structures (MSCS) [153]. In this method, small molecular probes, namely, organic solvents with hydrophobic to hydrophilic properties that vary in size and shape, are placed on a dense grid around the protein (16 solvent molecules). Each probe molecule generates many bound positions on the protein surface using rigid body docking with the fast Fourier transform correlation approach. For each probe, billions of docked conformations are sampled, eventually, the 2000 best poses for each probe are listed for further processing. The minimized probe conformations are clustered based on root mean square deviation (RMSD), and hot spots are defined by RMSD and consensus clustering. The binding site that has largest cluster is defined as one hot spot. The druggability of hot-spots method was applied to well-known PPI complexes such as IL-2IL2R, Bcl-xL, p53-MDM2, and TNFα-TNFR1.

**Table 4:** A list of software tools and servers available for identification of hot spots.

Method	Website	Type	Reference
FTMap	http://ftmap.bu.edu/login.php	webserver	[150]
PredHS	http://www.predhs.org/	webserver	[154]
HSPred	http://bioinf.cs.ucl.ac.uk/structure/	webserver	[155]
iPred	http://modlab-cadd.ethz.ch/software/ipred/	Java based webtool	[156]
HotPoint	http://prism.ccbb.ku.edu.tr/hotpoint/	webserver	[157]
ROBETTA	http://robetta.bakerlab.org/	webserver	[158]
KFC2	http://kfc.mitchell-lab.org/	webserver	[159]
PCRPi	http://www.bioinsilico.org/PCRPi/	webserver	[160]

Most of the websites that are related to these software are linked via http://www.predhs.org/.

Protein-protein interactions can also be viewed as protein-peptide interactions as a small fragment of one protein interacts with protein surface of another protein. The peptide part that binds could have a particular secondary structure or a linear chain. Examples of such protein-peptide interactions include SH3 domain, WW domain (WW domain, one of the smallest protein modules, is composed of 40 amino acids and mediates specific PPI with short proline-rich or proline-containing motifs), and PDZ domain [161,162,163,164,165]. Most of the protein-protein docking software may not be useful for peptide-protein docking as protein-protein docking software does not incorporate flexibility of side chains of both partner molecules. Software that is used for small molecule-protein docking has limitations in terms of the number of rotatable bonds for flexibility. London *et al.* [162] have developed a method that can be applied to peptide-protein docking. This method uses a coarse model of interaction to start with and Monte-Carlo simulations to refine the model using energy minimization. The model generated using this method refines the backbone and side chains of proteins as well as peptides in bound form, and the structure of the complex obtained is of relatively high resolution.

Based on hot spots and the structure of the protein complex, compounds that bind to one of the protein partners and inhibit PPI are designed. Structure-based design or database screening is used to arrive at a hit compound and a template structure is designed. Once the basic template structure of the compound is designed and synthesized, one has to show experimental evidence that the designed molecule is indeed a PPI inhibitor of particular partner proteins. Results obtained from docking studies should be validated using experimental methods. Several methods are available to study PPI and their inhibition. Since the intent of this article is to provide an overview of docking methods in the design of PPI inhibitors or modulators, we have briefly highlighted some biophysical methods that are used in concert with docking methods to validate the hit compounds. Surface plasmon resonance (SPR) [166], and NMR techniques can be used to provide the information about binding of small molecule/peptides to a protein of interest. These methods require the protein to be in the pure form, and the binding information obtained is direct evidence of binding of a designed molecule to the target protein. SPR methods provide detailed information about kinetics of binding of small molecules to proteins. NMR is used widely to obtain SAR to design the inhibitors of PPI [167]. Such a method is useful to screen a large number of small molecular fragments to bind to one subsite of a protein surface [168]. *In vitro* methods such as proximity ligation assay [169] and enzyme fragment complementation assay [170] provide dimerization of proteins and inhibition of the dimerization of proteins by small molecules. Other experimental techniques such as FRET [171], and mass spectrometry are also available for studying PPI. Examples of the use of such methods are available in recent literature [42,172,173,174,175,176].

### 3.2. Examples of Design of PPI Inhibitors

Once the hot spots are identified on the protein surface, molecules that are designed to inhibit PPI can be docked to the protein with a grid box covering the area of several hot spots, and the binding ability of the designed compound to one of the proteins can be evaluated. At this stage, any of the docking methods that evaluate small molecule or peptide docking to proteins can be used. The most widely used methods are AutoDock [177], DOCK [178], FlexDock [179], Glide [180] and others [12,181]. In the design of PPI inhibitors there are only a few categories. These are antibodies that inhibit PPI, peptides and peptidomimetics that inhibit PPI, and small molecules that inhibit PPI. We will not cover any aspects of antibody-based PPI in this article. Among the small molecule-based PPI inhibitors, the design concept may start with peptides that may later be converted into peptidomimetics and still later into small organic molecules. When molecules are designed based on the protein surface of one of the interacting protein partners, the design is based on certain protein recognition motifs. It is known that certain molecular scaffolds are associated with exhibiting biological or pharmacological activity when incorporated into drug design. Secondary structures such as α-helix [182,183,184], β-sheet [185], or β-turns [186,187] often provide structural scaffolds for the design of inhibitors of PPI. Extended structures and proline-rich segments [188] also form motifs for design. Organic molecules such as benzodiazepines are good scaffolds for protein recognition sites [189]. Such molecular scaffolds are also called “proteomimetics” [190,191]. Here we will illustrate some case studies of PPI inhibitors. These PPI inhibitors could be small organic molecules or peptides or peptidomimetics. Some examples start with peptides that are later modified to organic molecules based on the structures of the important functional groups. The discovery of PPI inhibitors has several well-known examples such as p53-MDM2 inhibitors, IL2-IL2R inhibitors *etc.* However, the design in these examples includes fragment-based discovery [192] and experimental screening techniques using SAR by NMR [151,193] to screen several compounds to obtain the hits. Such methods are described in several reviews [35,36,37,47,194,195]. Some recent reports of the design of PPI inhibitors that incorporates the docking method include the design of NF-κB antagonist [196], a TNF-like weak inducer of apoptosis (TWEAK) and fiboblast growth factor inducible 14 (Fn-14) [197], toll-like receptor 4 (TLR4) and myeloid differentiation factor 2 (MD-2) [198], calmodulin (CaM) and smooth muscle myosin light-chain kinase (smMLCK) [199], and EphA2-ephrin-A1 [200]. Here, we will focus on some recent examples of PPI targeted for drug design that were reported in the literature as well as PPI inhibitors designed in our laboratory.

**Figure 2:**
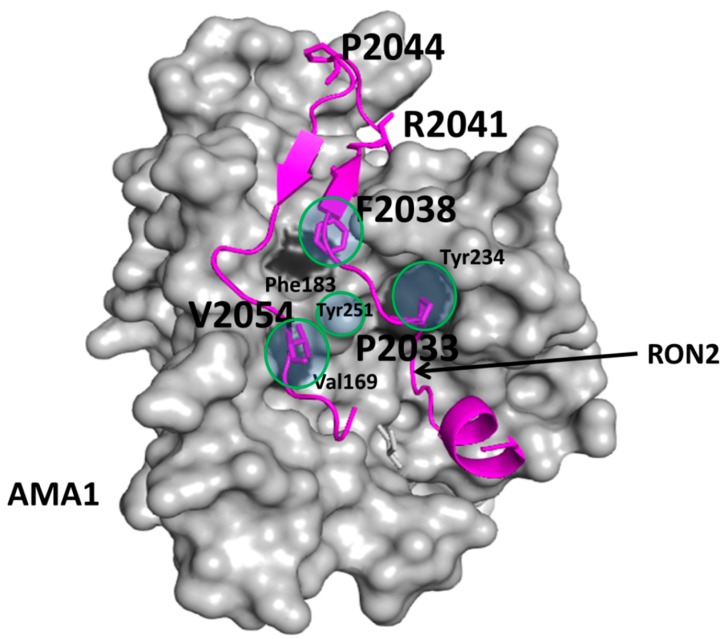
Crystal structure of complex of AMA1-RON2 peptide (PDB ID:3ZWZ). AMA1 is shown as the surface and RON2 is shown in magenta. Amino acids from AMA1 that are critical for binding are labeled with three-letter codes. Amino acids from RON2 that are critical for binding and used in the design of the pharmacophore model are shown as sticks (magenta) and labeled with single letter codes for amino acids. Four point pharmacophore (shown with circles) was generated based on the PPI interaction. However, Tyr251 was in the exclusion sphere in the pharmacophore generated [204]. PyMol (Schrodinger LLC, Portland, OR, USA) was used to generate the figure based on the information from Pihan *et al.* [204].

Much effort has gone into designing new antimalarial agents since most of the antimalarial drugs that were marketed decades ago have shown resistance to malarial parasite *plasmodium falsiparum*. The PPI inhibition strategy was used to discover new antimalarian agents. Invasion of red blood cells by plasmodium parasite involves the interaction of two proteins, apical membrane antigen 1 (AMA1) on the surface of the parasite and Rhoptry neck protein 2 (RON2) that is discharged from the parasite and imbedded in the membrane of the host cell [201]. Peptides derived from RON2 were able to compete with the binding interaction of RON2 with AMA1 [202]. A small molecule screening HTS assay resulted in a molecule that had weak binding affinity and unfavorable properties for drug development [203]. Starting from peptides, different small molecules and peptidomimetics were developed. Pihan *et al.* [204] have used biophysical as well as computational methods to find potent PPI inhibitors of the AMA1-RON2 interaction that can be used as potential antimalarial agents. The structure of the complex revealed that RON2 peptide (amino acids 2021 to 2059) was buried in a hydrophobic grove with a surface area of 1700 Å^2^. Using mutagenesis studies, hot spots on the interacting proteins have been elucidated. On AMA1, Phe183 and Tyr234 had an effect on binding upon alanine mutation (Figure 2); for RON2, Pro2033, Phe2038, Arg2041, and Pro2044 were critical in binding to AMA1. Using these hydrophobic residues, a pharmacophore was created using different databases (Cambridge’s EXPRESS pink collection and MMsINC). After initial ligand-based step screening, docking was used to further filter the compounds generated based on the pharmacophore. The software PLANTS [205] was used to dock the generated molecules to the protein complex of AMA1-RON2. Here, docking was used to filter a large number of compounds as possible PPI inhibitors from the pharmacophore modeling and database screening. For the final selection, compounds that dock to the AMA1 groove and interact with key residues Tyr234, Phe183, and Val169 were selected. Only eight compounds that mimicked the Phe2038 binding were selected for biological assay and binding studies using surface plasmon resonance and biophysical studies.

A challenging PPI inhibitor design was reported by Broos *et al.* [206] in which the proteins that interact change the conformation upon binding. In such cases, the inhibitors may not bind to the PPI interface, rather, they bind near the PPI interface. The von Willebrand factor (VWF) that is important in thrombosis undergoes conformational change due to shear stress conditions, allowing platelet glycoprotein (GP) Ib-V-IX to bind to the VWF-A1 domain. The formation of this complex is important in thrombus formation; thus, the inhibition of this complex is important in the development of antithrombotic agents. Monoclonal antibodies and nanobodies have been developed as antithrombic agents [207,208]. However, because of the limitations of antibodies as drugs, there is an interest in the development of small molecules. The crystal structures of the free VWF and complexes of VWF-GP-Ib-V-IX [209] as well as data from mutational studies were used to design a small molecule to inhibit the PPI of VWF and GP-Ib-V-IX.

**Figure 3:**
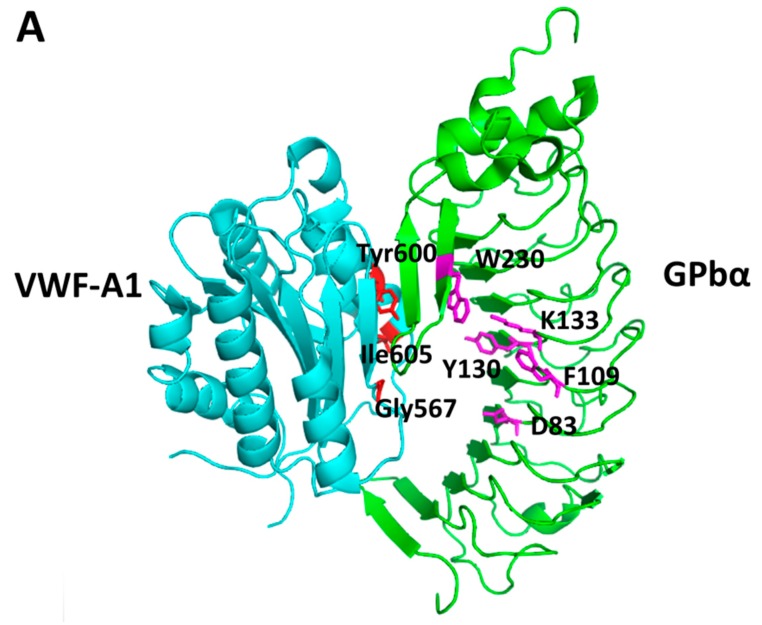

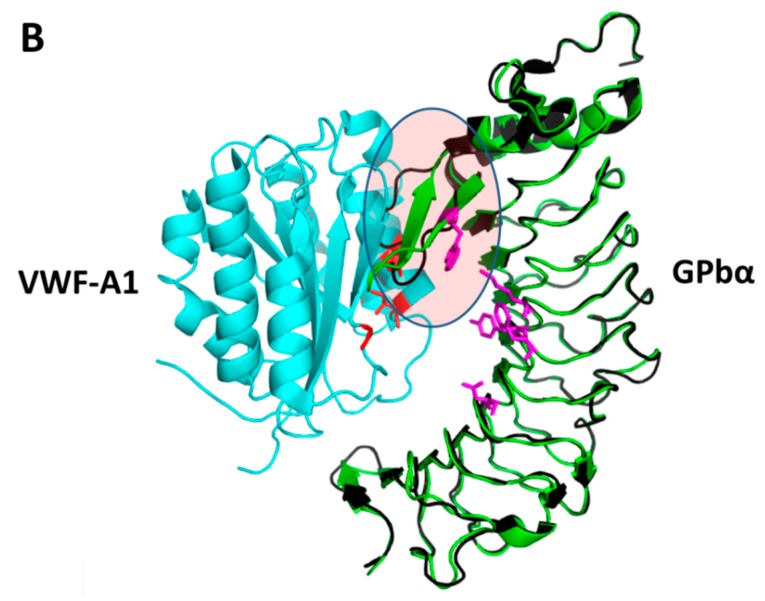
(**A**) Crystal structure of the complex of VWF-A1 and GPIbα (PDB ID: 1SQ0) [209] showing the hot spots determined by computational methods. Residues in the hot spot region of VWF-A1 are shown as red sticks. Residues from the hot spots of GPIbα are shown as sticks in magenta. Amino acids from VWF-A1 are labeled with three-letter codes and those from GPIbα are labeled with single-letter codes; (**B**) Crystal structure of the complex of VWF-A1 and GPIbα (PDB ID: 1SQ0) [209] overlapped with the unbound structure of GPIbα (PDB ID: 1P9A) (shown in black). Note the marked oval shape where there is a change in the conformation between the free and bound states of GPIbα. This region is the most probable hot spot on the protein to be involved in binding of a PPI inhibitor [206]. PyMol (Schrodinger LLC) was used to generate the figure based on the information from Broos *et al.* [206].

Crystal structures (Figure 3) were used to identify the hot spots using PASS [210], Hotpatch [211], and site Finder algorithms. Furthermore, molecular dynamic simulations were carried out on the selected crystal structures of VWF-A1 and GPIbα to evaluate the stability of hot spots. This step is particularly important as VWF undergoes conformational change during binding to GPIbα. Those hot spots that were stable during MD simulations were considered for binding of drug-like molecules. The two pockets selected were pocket 1 on GPIbα, with amino acids Asp83, Phe109, Lys133, Tyr130, and Trp230 and pocket 2 on VWF-A1 consisting of residues Gly567, Ile605, and Try600. Using the molecular databases Asinex and Enamine nearly 1,500,000 compounds were selected, and the databases were filtered using SMPPII-like criteria to obtain a subset of approximately one-million compounds. Next, using MOE, the database was reduced to 80,000 compounds. These compounds were screened by docking the compounds on VWF-A1 and GPIbα using GOLD. Finally, 24 molecules were selected from the docking studies for *in vitro* testing. The small molecules exhibited platelet aggregation activity at micromolar concentration, and saturation transfer difference (STD) NMR studies indicated binding of these small molecules to GPIbα.

**Figure 4:**
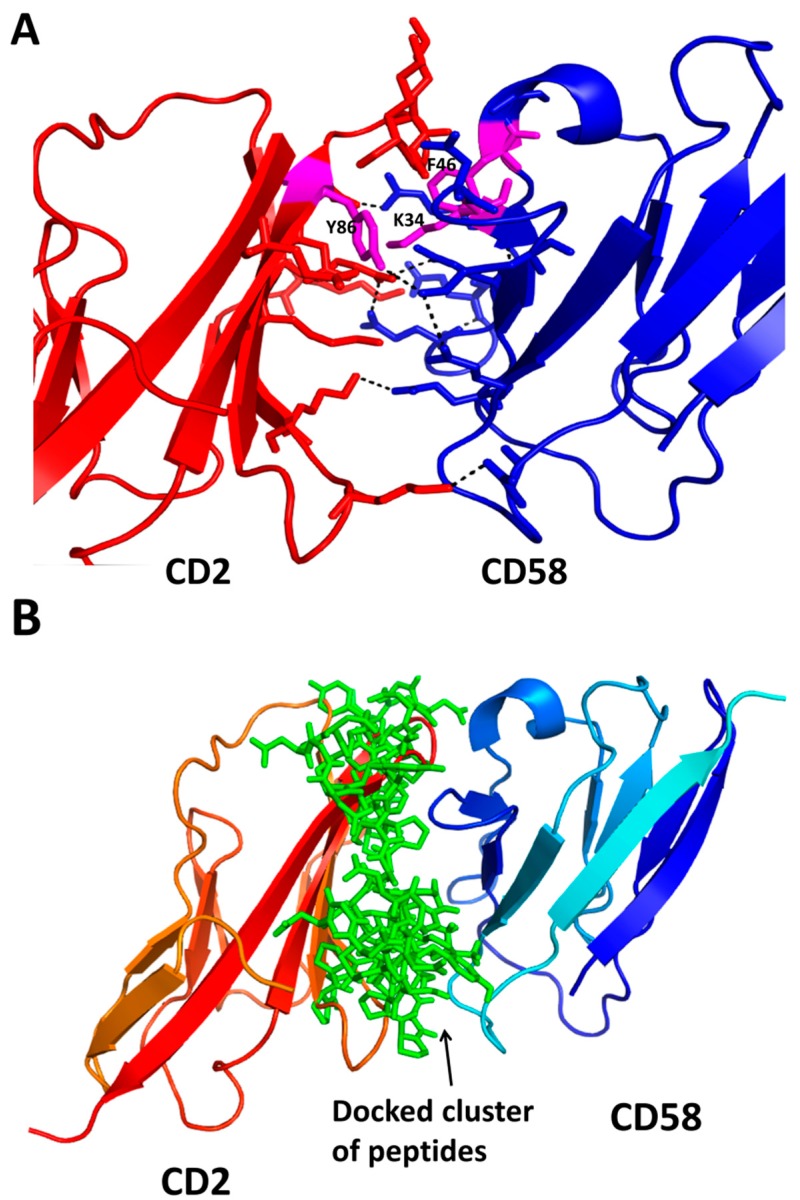
(**A**) Crystal structure of the complex of CD2 and CD58 (PDB ID: 1QA9) [217] adhesion domain involved in the recognition of T cells and antigen-presenting cells. Salt bridges and hydrogen bonds are shown by dashed lines. The hydrophobic hot spot is shown as magenta colored sticks. Note that, although the PPI surface is dominated with salt bridges and hydrogen bonds, the hydrophobic interaction between Tyr86, Phe46, and the Lys34 side chain sandwiched between the aromatic residues forms the hot spot; (**B**) A cluster of low-energy docked structures of a peptide bound to the CD58 protein adhesion domain [218,219,220]. PyMol (Schrodinger LLC) was used to generate the figure.

As mentioned earlier, most of the PPI inhibitors are based on the structure of one of the protein partners and, hence, peptides designed from the interface are natural PPI inhibitors. In our lab, we use peptides and peptidomimetics as PPI inhibitors. The T-cell adhesion molecules or co-stimulatory molecules CD2-CD58 have an important role in the immune response in strengthening the adhesion of T cells to antigen-presenting cells (APC). The protein-protein interaction between CD2 on T cells and CD58 on APC is important in the early stages of immune response [212,213,214]. Blocking adhesion/co-stimulatory molecules results in obstructing the T-cell receptor-APC interaction and preventing the primary immune response. CD2 and CD58 molecules have been shown to be important in inflammatory and autoimmune diseases such as rheumatoid arthritis (RA) [215]. In rodents, CD48 is involved in interaction with CD2 for immune response similar to that with CD58 in humans. Crystal structure and mutagenesis studies are available for CD2-CD58 interaction [216]. The CD58 binding domain of CD2 consists of β-strands F and C with charged residues (Figure 4A). A glance at the interfacial structure of CD2-CD58 proteins indicates that electrostatic interactions dominate the interface region with 10 salt bridges and five hydrogen bonds [217] and the interface area is around 1200 Å^2^. However, mutagenesis studies indicated that mutation of the residues involved in salt bridges and hydrogen bonds did not affect the binding of two proteins significantly. Mutation at a hydrophobic Tyr86 reduced the binding affinity of CD2-CD58 by 1000 fold, suggesting that the hot spot is a hydrophobic region. Our idea is to design peptides to mimic the CD58 binding region of CD2 protein that blocks CD2-CD58 interactions. We have designed several peptides and peptidomimetics that bind to CD58 and block the CD2-CD58 interaction [218,219,220,221]. The designed molecules were docked to CD58 as well as CD48 to understand the nature of the interaction between the ligand and the receptor (Figure 4B). *In vitro* and *in vivo* studies suggested that the designed peptides suppress the adhesion between T cells and antigen-presenting cells by inhibiting CD2-CD58/CD48 interaction and are able to suppress immune response in an autoimmune disease model of rheumatoid arthritis.

The literature suggests that PPI hot spots have some common features; however, PPI surfaces in different proteins are different, which makes the interactions highly specific. When homologous protein partners are involved, how do we design PPI inhibitors that bind specifically to one protein and inhibit PPI interaction? We have studied such cases in our laboratory using experimental and computational approaches.

**Figure 5:**
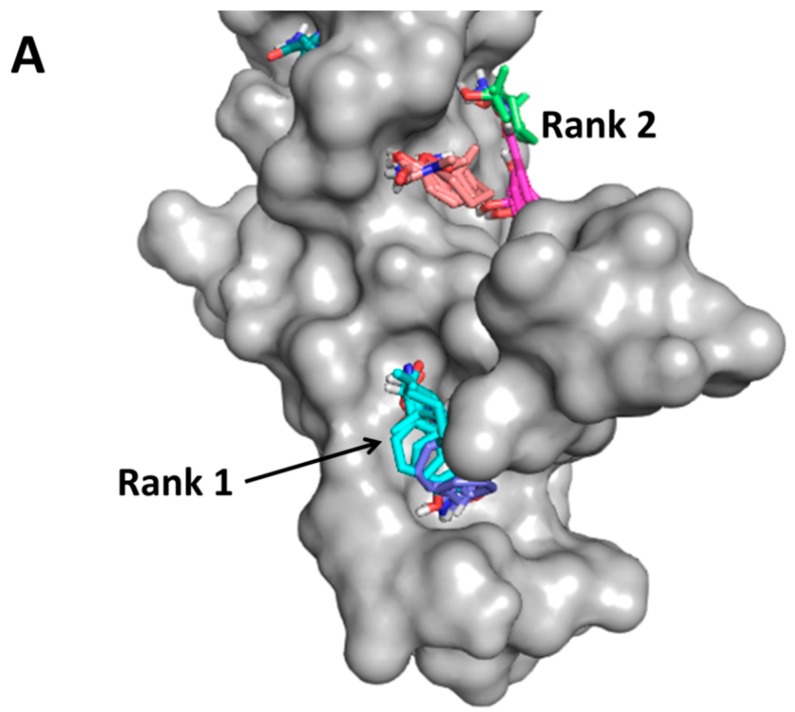

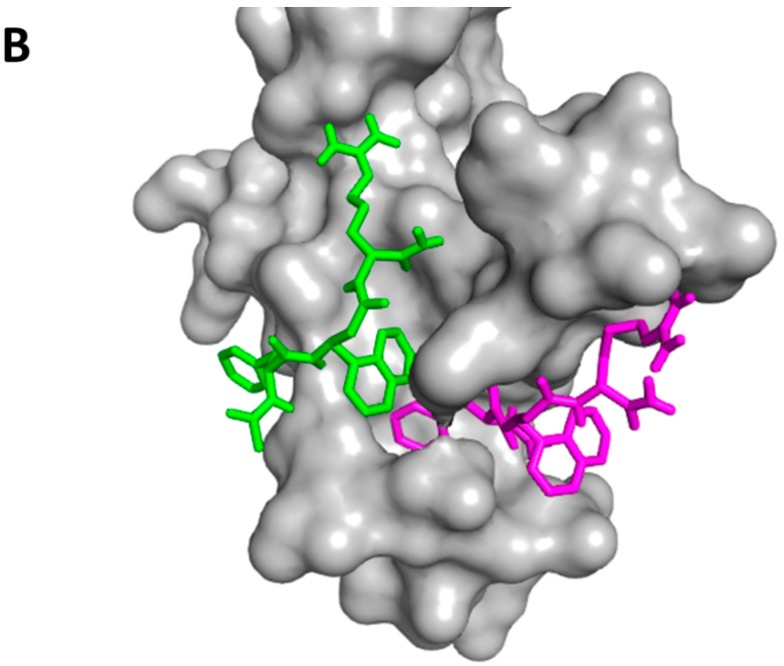
Crystal structure of the HER2 protein (PDB ID: 3N85) [233] extracellular domain IV shown in surface representation. (**A**) Hot-spot region on the surface was identified by FTMAP [150]. Two hot spots were identified; (**B**) Docking of a peptidomimetic designed to bind to domain IV of HER2 protein. The peptidomimetic docked at rank 1 hot spot suggests that hot spots are suitable sites for the design of PPI inhibitors [231]. PyMol (Schrodinger LLC) was used to generate the figure.

The human epidermal growth factor receptor (EGFR/HER) system of receptor tyrosine kinases plays an important role in cell growth and differentiation [222]. Among these, EGFR-HER2 and HER2-HER3 dimers are well known in cancers. HER2 overexpression and its dimerization leads to an aggressive form of breast cancer [223,224]. Blocking of the extracellular domain IV of HER2 by the antibody trastuzumab is known to be clinically significant [225]. Our strategy is to use peptidomimetics to inhibit protein-protein interactions in the key regions of the interactions. We have designed a small molecule (a peptidomimetic) that has been shown to bind to HER2 domain IV and modulate dimerization of EGFRs. All four EGFRs have an overall similar 3D structure and nearly 50% sequence similarity. The molecule we have designed binds only to the HER2 protein extracellular domain and inhibits PPI of EGFR:HER2 and HER2:HER3 heterodimers that have important implications in breast, lung, and ovarian cancers. Using docking studies, we have defined the possible binding site of the molecules on the PPI interface of HER2 protein (Figure 5A,B). Using a variety of experimental methods, the molecules designed were shown to inhibit PPI, and these molecules were able to suppress tumors in a xenograft model of breast cancer [226,227,228,229,230,231,232].

Most of the PPI studies and docking methods describe a particular set of PPI and finding inhibitors for that particular PPI using docking methods. Casey *et al.* [234] described a method of combined normalization of ligand and target, which resulted in improved ability to rank true positives of multiple ligands binding to multiple protein surfaces. Usually in docking, scoring and ranking are relative and applicable to the case of many ligands and one protein target. When we want to compare many ligands binding to different proteins, the ranking methods do not provide the best results because scoring and ranking are relative. In the method developed by Casey *et al.*, the results are normalized in both ligand and target dimensions. This method was applied to dock 287 FDA-approved small drugs with 35 small peptide-binding proteins, which include 15 true positives. AutoDock 4.0 utility was used as a docking function. Different known targets and their ligands such as ML-IAP/XIAP, Factor VIIa, Renin, Bcl-2, Thrombin, and MDM2 were used for virtual normalization. The results suggested that the method provides true positives (molecules that actually inhibit PPI in experimental studies). Such normalized methods are very useful for creating a diverse set of compound data bases that can be used to screen PPI inhibitors for any given PPI. Such docking methods need high-performance computers with parallel processors and computational methods applicable for parallel processing [234].

**Figure 6:**
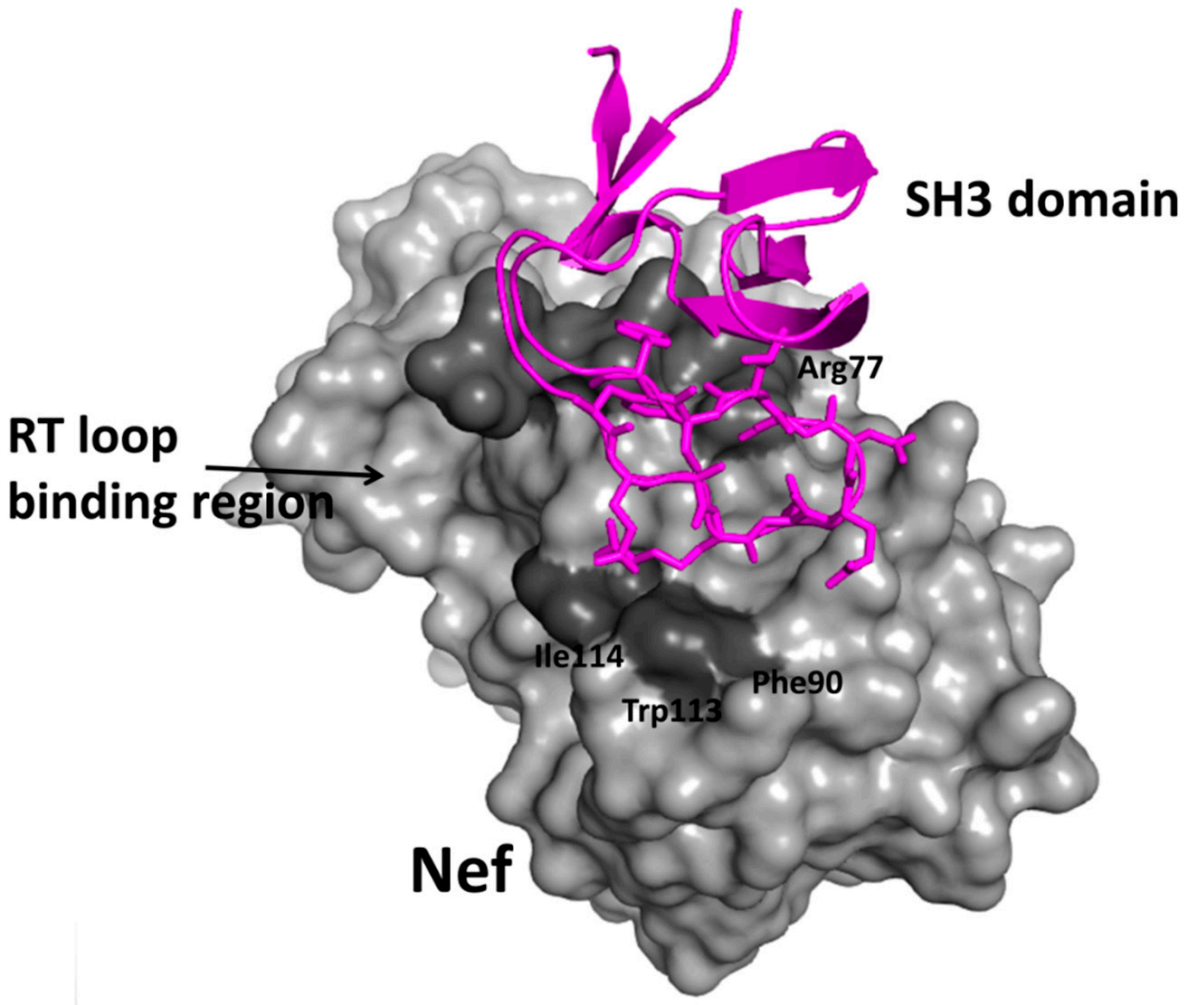
Crystal structure of HIV-1 nef-SH3 domain (PDB ID:1AVZ). Nef is shown in surface representation and SH3 domain in magenta. Note the interaction of SH3 domain with hydrophobic grove that is called RT loop binding region. Residues surround the groove are shown in dark shade. Compounds were screened based on RTloop binding region [235]. PyMol (Schrodinger LLC) was used to generate the figure based on the information from Betzi *et al.* [235]. Permission obtained. Copyright (2007) National Academy of Sciences, U.S.A.

In HIV-1 infection, many active protein-protein interactions between host and pathogen play a vital role. HIV-1 Nef is one of the important proteins involved in infection, pathogenicity, and disease progression. Nef proteins have well-defined Src homology 3 (SH3) binding surface through which interactions are formed (Figure 6). Betzi *et al.* [235] made a successful attempt to target the SH3 binding site on HIV-1 Nef protein to inhibit HIV-1 Nef/SH3 protein-protein interaction by rational drug design. A combination of virtual screening, high-throughput screening, and *in vitro* filters was used for the drug design. From the NCI diversity library (https://dtp.cancer.gov/branches/dscb/div2_explanation.html) of drug-likeness criteria, 1420 molecules were retained on which high-throughput docking was performed. FlexX software was used for virtual high-throughput screening, and calculated scores were reevaluated by GFscore. The first 335 lowest energy compounds were selected from docking. This selection was further narrowed by applying a filter based on RT loop binding region of SH3 domain (Figure 6). The final 10 compounds were tested in *in vitro* cell-based assays; this resulted in two potent drug-like hits that can inhibit the HIV-1 Nef/SH3 interaction competitively. The study is a clear example that the *in silico* approach can be used to design a small molecule PPI inhibitor [235].

Using a docking approach, Nomme *et al.* [236] designed a peptide that inhibits HsRAD51-ssDNA interaction. Homologous recombination is one of the important DNA repair processes. RAD51 protein is involved in the repair of a double-stranded break, which is the most severe DNA damage. Rad51 is overexpressed in various cancers, which causes resistance to the anticancer treatment. The amount of overexpression of Rad51 correlates with resistance to cancer treatment as well as degree of cancer advancement. Breast cancer (BRC) motifs of human BRCA-2 are involved in the interaction with human RAD51 (HsRAD51), and are reported to inhibit the filament formation of HsRad51. A small 28-amino acid peptide designed from BRC motifs was previously found to be a potent inhibitor of HsRAD51 *in vitro*, but was not efficient for medical use. Using a docking approach, a chimera peptide was designed based on eight existing human BRC protein motifs. To design the peptide, models of all eight BRC motifs were complexed with HsRad51 on the basis of HsRad51-BRC4 motif crystal structure. The complex modeled was analyzed for interaction energy of each amino acid residue of the BRC motifs and the best amino acid residue in the binding position with HsRad51 was selected. The designed chimera peptides were evaluated for their PPI inhibition potential. One of the chimeric peptides designed exhibited 10 times more potency than a similar peptide described in previous studies. The study demonstrates that PPI inhibitors can be designed when crystal structures of homologous proteins are known [236].

Protein-protein interaction can also be stabilized by drug-like molecules. These PPI stabilizers can bind to one partner protein and stabilize the interaction of the two proteins (allosteric effect) or bind to the interface of the complex, stabilizing the dimers. Such examples are available in the literature, and the molecules that bind to stabilize the complexes are already on the market. One of the best examples of protein stabilizers is paclitaxel, which binds to microtubules and stabilizes the protein dimers [14,237]. Here, we provide one example of identification of a PPI stabilizer using a docking method.

Myc is similar to a myelocytomatosis viral oncogene that codes for a transcriptional factor oncoprotein Myc. Myc shows constitutive overexpression in many types of cancer and, hence, is a target for cancer therapy. Myc protein belongs to the helix-loop-helix leucine zipper protein family that activates or represses transcription as a heterodimer. The heterodimer partner of Myc is myc-associated factor X (Max). Max also belongs to the helix-loop-helix leucine zipper protein family, and can form homo- and heterodimers. Both Myc and Max are needed in heterodimerization form to bind DNA since Max lacks a transactivation domain and Myc contains a transactivation domain but cannot homodimerize. Myc can heterodimerize with Max to form heterodimers that can both bind DNA and transactivate. The transcriptionally active Max/Myc dimer promotes cell proliferation as well as apoptosis. Overall, Myc-Max heterodimers promote cancer and Max homodimers suppress cancer [238]. This PPI inhibition of Myc-Max has therapeutic potential for cancer. Small molecules that inhibit PPI of Myc-Max have been reported [16]. Jian *et al.* [238], have used a novel way to find therapeutic agents using a different strategy to reduce the Myc-Max heterodimer. The idea was to stabilize the Max-Max homodimers so that Max is not available for heterodimerization of Myc and downregulation of the entire Myc network. Furthermore, Max-Max homodimers suppress the cancer; thus, stabilization strategy is a new way of finding therapeutic agents. The crystal structure of the Max-Max homodimer is available [239] and, hence, the dimer structure can be used for docking. The PPI surface of the Max homodimer was studied, and stabilizers of Max homodimers were achieved via virtual screening using AutoDock. The authors report the screening of 1668 compounds from a NCI diversity set against Max homodimer as well as Myc-Max heterodimer using a blind docking method. By analyzing the different binding sites, the authors identified the compounds that are specific to Max homodimer stabilizers. Overall, this type of method needs more computational time since the entire binding area in PPI has to be explored on docking for identification of PPI stabilizers.

PPI inhibition for designing drug-like molecules is an area that has been explored extensively during the last decade. Considering the number of databases available for PPI inhibitors and the limited number of 3D structures available for proteins, docking and scoring methods play a major role in various stages of screening and designing PPI inhibitors as well as stabilizers. Starting from protein-protein docking, binding site analysis, and screening compounds for drug-like compounds, docking methods are used for identification of hot spots and design of PPI inhibitors. There are three major challenges in the use of docking on relatively flat surfaces of PPI. The first problem is the flexibility of proteins in docking and scoring. This is particularly important when the protein-protein interactions are transient. Furthermore, if there are conformational changes in the binding site in the bound or unbound state of one or both of the proteins, docking methods have to incorporate a method to deal with this. The analysis of ensembles of conformations and prediction of consensus hot spots is a good option for proteins that have a flexible surface. The second major problem is taking into account the interaction of small molecules or peptides with water molecules in the bound state. Since the PPI surface is flat, even if the hot spots are hydrophobic, only one part of a small molecule or peptide is buried in the protein shallow groove or cleft. The part of the ligand molecule that binds to the surface of the protein may not have any contact with water molecules. In most of the PPI hot spots, water molecules often form a ring around the center of the hot spot, and the hot spots are dry. Hence, the contribution of water molecules to the energetics of binding is small [20]. The other part is exposed to solvent if the protein of interest is an extracellular or cytoplasmic domain. Although some docking programs can incorporate water molecules, most of the applications are related to enzyme-active sites where interaction with water molecules within the enzyme cavity is well defined or there are a limited number of water molecules within the cavity. With limited data on protein complexes and the PPI inhibitors, inclusion of water molecules is still questionable and depends on each case studied. The third major challenge in PPI is protein-protein docking in the case of the absence of 3D structure of the complexes. It is almost impossible to search for all possible interactions between two proteins with different orientation and rotation. Most of the protein docking results should be evaluated based on experimental data.

A promising area in PPI inhibitors is fragment-based drug design, in which fragments are developed on the PPI and linked to create new PPI inhibitors. Docking will play a significant role in this method also. There are attempts to create focused libraries for specific target types. Such PPI inhibitor libraries can be used to dock compounds using virtual screening. Rather than screening the entire data set (for example, natural product compounds, NCI), employing only a limited data set of compounds that are useful for target protein surfaces is more efficient [240]. A data set of PPI inhibitors would be useful for docking the known diverse PPI inhibitors because a traditional data set of inhibitors may not be useful for PPI inhibitor screening. A study was conducted to compare high-affinity inhibitors of IL-2, Bcl-XL, HDM2, and HPV E2 with compounds in the databases MDL Drug Data Report and World of Molecular Bioactivity (WOMBAT) by using the compounds’ Similarity Ensemble Approach. The results of the study did not show any similarity between the PPI inhibitors used and the compounds in the database [26]. To screen PPI from chemical databases of compounds, a very large collection of data sets may be needed. However, small molecular databases can be used to screen fragments in the fragment-based approach.

## 4. Conclusions

The design of PPI inhibitors is gaining confidence as more molecules that have drug-like properties are generated. Without defining the proper surface for PPI, the design of molecules that inhibit PPI will be vague. Docking methods are used in various stages of PPI inhibitor design whether it is small molecule-based or peptide-peptidomimetic-based drug design. Improved docking methods are necessary as a starting point for PPI inhibitor design. A combination of experimental and computational techniques with scoring functions appropriate for particular cases is necessary for designing PPI inhibitors. Docking algorithms used for PPI are in the early stages; however, as more data are available, it will become a highly promising area in the design of PPI inhibitors or stabilizers.
